# Medical decision making for patients with Parkinson disease under Average Cost Criterion

**DOI:** 10.1186/1743-8462-6-15

**Published:** 2009-06-24

**Authors:** John E Goulionis, Athanassios Vozikis

**Affiliations:** 1 Department of Statistics and Insurance Science, University of Piraeus, 80 Karaoli & Dimitriou Street, 18534 Piraeus, Greece; 2 Department of Economic Science, University of Piraeus, 80 Karaoli & Dimitriou Street, 18534 Piraeus, Greece

## Abstract

Parkinson's disease (PD) is one of the most common disabling neurological disorders and results in substantial burden for patients, their families and the as a whole society in terms of increased health resource use and poor quality of life. For all stages of PD, medication therapy is the preferred medical treatment. The failure of medical regimes to prevent disease progression and to prevent long-term side effects has led to a resurgence of interest in surgical procedures. Partially observable Markov decision models (POMDPs) are a powerful and appropriate technique for decision making. In this paper we applied the model of POMDP's as a supportive tool to clinical decisions for the treatment of patients with Parkinson's disease. The aim of the model was to determine the critical threshold level to perform the surgery in order to minimize the total lifetime costs over a patient's lifetime (where the costs incorporate duration of life, quality of life, and monetary units). Under some reasonable conditions reflecting the practical meaning of the deterioration and based on the various diagnostic observations we find an optimal average cost policy for patients with PD with three deterioration levels.

## Introduction

Parkinson's disease (PD) is characterized by a progressive loss of substantia nigra pars compacta (SNc) neurous of unknown etiology [1]. The gold standard for PD diagnosis is the neuropathological examination. Since there are no known clinical biomarkers for disease detection, diagnosis is based on clinical criteria. The three main features are tremor, rigidity and motor dysfunction such as freezing and bradykinesia (slowness of movement). In the early stages of PD, symptoms and signs are asymmetrical however, with disease progression, PD becomes a bilateral condition. Although major symptoms can be attenuated by dopaminergic medication within the first years of PD, no treatment is currently available to stop or slow the on going nigral degeneration [2].

Clinical symptoms of PD may include from slowness in activities of daily living (ADL) (such as dressing, walking and doing household chores, difficulty and taking longer to get up from a chair, reduced arm swing, flexed posture and a shuffling gait (bradykinesia), rigidity, and cogwheeling (ratchet-like feel of muscles) on passive movement. The Unified Parkinson's Disease Rating Scale (UPDRS), is a measure of overall motor function evaluation. Also the Activities of Daily living (ADL) score may provide new elements for the same evaluation [3].

Treatments for PD aim to improve motor function and quality of life. Clinical management varies with disease severity and the age of patient. The severity of disease is defined as the degree of functional disability, whereas the age of the patient is important with respect to the adverse effects of the drug being prescribed, see [1]. During the last few years, Deep Brain Stimulation (DBS) of the Subthalamic Nucleus (STN) has emerged as a promising therapy, alleviating major motor symptoms of Parkinson's disease. Deep brain stimulation is a surgical procedure indicated in the relief of symptoms of Parkinson's disease, essential tremor and dystonia. It involves the surgical implantation of the DBS device, which includes the implantable pulse generator or stimulator, the extension, and the lead. The electric impulse is produced within the stimulator component, and transmitted to the brain site by the extension and the leads. DBS surgery can be either unilateral or bilateral. The laterality of the surgery and target area for brain stimulation may vary with the type of symptom or spectrum of symptoms and such decisions are made on a case-by-case basis.

In general, however, the target areas for DBS stimulation are as follows, with the accompanying symptom: Thalamic region-predominantly for tremor. Subthalamic region-for tremor, dyskinesia, bradykinesia, akinesia, speech difficulties, and freezing globus pallidus, internal segment region for diskinesia, tremor, rigidity, bradykinesia, and akinesia. The clinical effectiveness of STN-DBS has largely been demonstrated and was verified by assessing Unified Parkinson's Disease Rating Scale (UPDRS) motor scores [4].

POMDPs are models which provide a powerful framework for decision theoretic planning of clinical actions. The above finite and infinite horizon Markov decision processes fall into the broader class of Markov decision processes that assume perfect state information-in other words, an exact description of the system. Numerous researchers have been made for the type of MDPs with variable discount rate see e.g [5-12]. Tests provide a more accurate estimation of the true state of the patient, but are subject to the error of tests. Extensions of MDPs, called POMDPs have been developed to deal with imperfect information [6]. In our paper we present a very effective and efficient POMDP formulation to the treatment options for patients with Parkinson's disease.

The average cost criterion is a popular criterion for optimization of stochastic dynamical systems over an infinite time horizon. On the theoretical side Astrom [13] considered the discounted cost (DC) criterion in great details. Commonly used method for studying the problem of existence of solutions to the average cost dynamic programming equation (ACOE) is the vanishing-discount method, an asymptotic method based on the solution of the much better understood discounted cost problem [14,15]. It is well known, at least in the case of finite state and action models, that if the ACOE admits a bounded solution, then one such solution can always be obtained as the limit of a sequence of differential discounted value functions, as the discount factor tends to 1.

In section 2, we present the main clinical results for the PD. Current medical therapies, although effective initially, become less efficient over time. The STN-DBS is an effective treatment that considerably alleviates the severity of signs and symptoms and improves the health status of patients with PD. In section 3, of the paper the POMDP model is described and some assumptions are provided. Moreover we present the development of POMDP formulation to the problem of treating patients with Parkinson's disease, under Average Cost Criterion. PD is a both debilitating and costly. In section 4 we present the model implementation using clinical data of 150 patients from a major Hospital in Athens area. Relevant economic data were taken from the medical records and costs were derived from different Greek medical economic resources. Costs were calculated from the perspective of the healthcare provider.

### Protocols for Clinical Treatments decisions for Parkinson's disease

#### Patient Selection Criteria for Deep Brain Stimulation

The evolving evidence on the risks and benefits of DBS warrants the careful selection of patients for this procedure. Such selection is intended to ensure the identification of patients most likely to benefit from DBS in the presence of significant risks associated with the procedure.

The main criterion to determine if a patient with PD is eligible for DBS is sensitivity to L-dopa. Responsiveness to an L-dopa challenge test has been found to be a strong predictor of DBS outcome; thus, it remains the main criterion of eligibility for this surgery. The procedure for the L-dopa challenge and measures of responsiveness are outlined in a diagnostic and methods core evaluation tool called the CAPIT protocol (Core Assessment Program for Intracerebral Transplantation) [16]. This protocol was developed as a minimum methodological standard to enable common practices between centres in the selection of and evaluation of patients for, in this case, DBS.

The CAPIT protocol has 2 main sections. The first is a description of the L-dopa challenge to be followed in assessing responsiveness to the drug. The second portion of the CAPIT protocol has a description of outcome measures to be included in the assessment that are based on the UPDRS scale (version 3.0), a measure of overall motor function. The UPDRS is a questionnaire that includes sections on motor function, ADL, and percentage of the waking day spent in good/poor function.

#### Essential tremor

Essential tremor is the most common adult tremor disorder. Tremor is typically evident on both sides of the body. Tremor occurs during voluntary movement, which is distinguished from tremor-predominant PD with symptoms occurring only at rest. Furthermore, disease progression in essential tremor typically results in an increase in intensity of symptoms without a corresponding expansion of symptoms, in contrast to the expansion of symptoms in PD. This differential pattern in disease progression between the 2 diseases is the main reason for the implantation of a unilateral DBS device in essential tremor and a bilateral device in PD (i.e. it may be sufficient to reduce the tremor on the dominant arm in essential tremor, however, bilateral surgery may be necessary in some patients). Unlike PD patients with refractory disease, patients with essential tremor may stop drug therapy entirely if the medications are not working.

#### Primary Dystonia

Dystonia is considered a syndrome of different causes, and not a specific disease entity. The symptoms that characterize this syndrome are muscle contractions with twisting and odd posture. Primary dystonia, that which is idiopathic or genetically determined, is the most common form.

Therefore, either unilateral or bilateral DBS may be performed, depending on the laterally of symptoms. The primary target for neurosurgery in the mid-1970s was the thalamus, but with improvement of dystonic symptoms in PD following pallidotomy, the globus pallidus has become the brain site of interest for such symptoms.

### Model development and formulation

POMDP is an appropriate technique for modelling and solving such stochastic and dynamic decisions. We present the POMDP model formulation in order to find an optimal policy treatment for a patient with PD under an average cost criterion.

A POMDP is typically defined as a six parameter tuple. The six parameters together capture all aspects of medical decision making. A Partially observable decision process is:



• *S *is the set of physical state of a patient with Parkinson's disease. The states of a patient with PD are coded with the numbers 1,2,3. Hence S = {1,2,3}.

**1**: A patient has mild adverse events. The main risk factor for PD is increasing age, with only 5% to 10% of patients having disease onset the age 40. For patients with mild adverse events, pharmacotherapy is the usual action.

**2**: A patient has moderate events

**3**: A patient is down.

• At any given time period, the decision maker selects one of the following actions. The actions for the therapy treatment of a patient with PD are coded with the numbers 0, 1. Hence A = {0, 1} is the set of actions and *a*_*t *_is the decision at the stage t.

*α *= 0: is the Medical treatment with incomplete monitoring.

*Medical Treatment *for PD aims to improve motor function and quality of life. Clinical management varies with disease severity and the age of the patient. The severity of disease is defined as the degree of functional disability, whereas the age of the patient is important with respect to the adverse effects of the drug being prescribed [17].

The length of follow-up in these studies ranged from 2 to 6 years [18-20].

*α *= 1: The action of *Surgical Treatment*. Standard care for patients with advanced PD includes modifications in their drug regimen, and the possible introduction of drug holidays. Drug holidays are phases where drug therapy is eliminated and then reintroduced at possibly lowered doses. For patients with motor fluctuations that are not adequately controlled by drug therapy, surgical intervention in the form of DBS may be an option. Nevertheless, evaluation of patient eligibility for DBS surgery must follow specific guidelines and is best done within a multidisciplinary expert centre. Expert consultation indicates that about 10% to 15% of all patients with PD become candidates for DBS. (Personal communication with clinical expert, February 2005).

• *T*: *S *× *A *× *S *→ [0,1]: is the transition function, and represents the dynamic of the problem. Operating the patient with Parkinson's disease causes it to deteriorate statistically over time, and when the *Surgical Treatment *is chosen, the patient's state may be improved. This is reflected in the state transition probabilities, which are selected as follows. The state of the patient undergoes deterioration according to a stationary discrete-time Markov chain having a known transition law. The core process {x_*t*_, *t *= 0,1,2, ...} is a discrete-time Markov process.

Let *p*_*ij *_denote the 1-step transition probability from state *i *to state *j*.

. The transition matrix  is time invariant and shows the effect of the treatment upon the patient state. We apply the EM algorithm [21] to estimate the above transition probabilities. Alternatively we can apply cohort studies.

◦ Θ = {1,2,3}, a set of possible observations. The main observations (Motor function, including tremor, ADL) are coded with the numbers 1,2,3. At each time period, the state of the patient is not known and is monitored incompletely by some monitoring mechanism under *Clinical Examinations *(CE). (CE) are characterized by the fact that they yield the opportunity to observe one or more external state variables. The results of (CE) may supply a better idea of the true state of the patient, but are subject to the error of the tests. Clinical presentation of PD may include slowness in activities of daily living (ADL) such as dressing, walking, and doing household chores, difficulty and taking longer to get up from a chair; reduced arm swing; flexed posture and a shuffling gait (bradykinesia); rigidity; and cogwheeling (ratchet-like feel of muscles) on passive movement.

• The probabilistic relation between observation process z_t _and core process x_t _is given by the 3 × 3 time invariant observation matrix. R. It's assumed that the probabilistic relation between the state of the system and the outcome of the monitoring is prescribed by the following known conditional probability:

*r*_*iθ *_= Pr{the outcome of the monitoring is level *θ*|the system is in state i},

*i *= 1, 2, 3 and *θ *= 1, 2, 3.



• The cost structure considered here is as follows: *c*^*a*^(*i*), where *c*(*i*, *a*) is the scalar valued cost accrued, when the current state is *i *∈ *S *and action is *α *∈ *A*. The costs c(*i*, *α*) for various kinds of combinations of *π *∈ ∏ and *a *∈ *A *are 0 ≤ *c*(1,0), ≤ *c*(2,0), ≤ *c*(3,0), ≤ c(*x*,0)< ∞, ∀ *x *∈ *S*. The above are valid because in the early stage of Parkinson's disease patients' symptoms are markedly alleviated by dopaminergic therapy. However at later stages of the disease motor fluctuations and or dyskinesias may develop which result in a major disability and a considerable decrease in the quality of life of patients. The cost of therapy cannot be judged without also considering the outcome of therapy and cost-effectiveness analysis links these two measures explicitly. As outcome measurement we used the UPDRS to evaluate the clinical endpoint as well as the sickness impact profile to evaluate the health status following DBS. The cost-effectiveness of DBS are evaluated by calculating the incremental costs of patients treated with DBS against the drug costs at baseline using the UPDRS as an outcome measurement. Using a decision analytic model from a societal perspective with a one year time horizon [22] evaluated the immediate agents preferences or rewards (costs).

• *β *is the discount factor of future costs arised in every next step of the model. *β *∈ (0,1).

Although the state of the core process is not known with certainty, it is possible to calculate the probability that the patient is in a given state. In particular we define:



The vector *π*(*t*) = (*π*_1_(*t*), *π*_2_(*t*), ..., *π*_*N*_(*t*)) is called information vector, and the space of all such vectors,∏, is called the information space.

(3.1)

It is well known that *π*(*t*) is a sufficient statistic [14,5]. More precisely, *π*(*t*) summarizes all of the necessary information of the history of the process for choosing an action at time *t*.

Since control policies are based on the information vectors the POMDP can be recast as a completely observable equivalent MDP with a continuous state space as given by Sondik [5]. If the information vector at time *t *is *π *and an alternative *α *is selected, and if an output *θ *results, then the new updating information vector *π*(*t*+1) is given by *T*(*π*|*θ*, *α*).

By Bayes' rule.

(3.2)

 is the probability of receiving observation *θ *at stage *t *+1, given that *π*(*t*) and *α *is the action selected at stage *t*.

(3.3)

 be the diagonal matrix having  as its *j*-th diagonal term and zeros for all off-diagonal terms. Assuming 1 = *col *{1, ..., 1}.

The objective of a POMDP is to find an optimal policy among the admissible policies such that it minimizes a given performance index, typically the total expected discounted cost to be accrued over the infinite horizon, or the expected long-run average cost, conditioned on the a priori *π*(0). These costs are defined in terms of the state *x*_*t *_for each admissible strategy, *δ*, and information vector *ρ*(0) of the initial state by some performance index, see Appendix 1 for details.

We assume that *cα *= {*c*(*i*, *α*)},,  are stage invariant arrays.

### Model implementation and study outcome evaluation

In order to illustrate the solution procedure for the problem we present, we tested the model accuracy using clinical data from a cohort of 150 patients for a time of two years from a major public General Hospital in Athens Area. The parameters used in the study are given as follows. S = {1,2,3}, Θ = {1,2,3}, A = {0,1}, discount factor *β *= 0.9. The patient model evolves in the following manner: The uncertainty of the patient health state with PD arises from the inability to know exactly the level of deterioration under clinical examinations and diagnostic procedures because we have the error of tests Therefore the state of the patient is partially observed, an action is taken, a reward is received (or cost incurred), and the patient transitions to a new state according to a known probability distribution.

The transition matrices are the following for *α *= 0 and *α *= 1 respectively:



The observation matrices are the following for *a *= 0 and *a *= 1 respectively:



Hence,





In this portrayal, the space of possible *π *vectors with three components is represented by an equilateral triangles, with each point in the triangle corresponding to a possible state (belief state). For each information vector *π *= (*π*_1_, *π*_2_, *π*_3_), the perpendicular distance from the point to the side opposite the i-th vertex is just equal to *π*_*i *_(*i *= 1,2,3). Thus, points closer to the *i*-th vertex correspond to states of information in which the process is believed more likely to be in state *i*.

The study estimated direct healthcare costs from the perspective of the Greek statutory health insurances. The expenses for drugs were calculated according to official Greek Price Lists. Greek health insurance systems reimburse the costs for outpatient care with a flat rate for each quarter, irrespective of continued revisits during the quarter. Thus, costs for out patient care were calculated by multiplying the flat rate for each quarter by the number of quarters that the patient could have presented several times a quarter in the outpatient clinic while the health insurance only reimbursed the flat rate.



**Figure 1:**
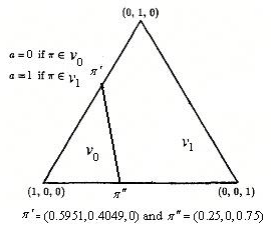
**The optimal policy**.

The initial information vector is *π*(0) = (0.4,0.5,0.1). The algorithm is a novel of a policy iteration. We begin with a policy δ^0^, evaluate that policy by solving a set of linear equations to find the value of this policy . Use this value to choose the action that minimizes the equations in (2.10) to perform a policy improvement step, and determine the next policy δ^1^. This process is continued until identical policies are found in subsequent iterations Sondik [5]. Optimal control-limit policy: A line segment connecting information vectors *π*' = (0.5951, 0.4049,0) and *π*" = (0.25, 0,0.75). For all information states (belief-states) to the right region we take surgical treatment. Otherwise we take medical treatment (see Figure 1).

Several studies have shown that dopaminergic medication can be considerably reduced after STN-DBS [23,24] Suggesting that costs for pharmacological treatment should also decrease. Also it has been demonstrated that nursing home admissions were less frequent in patients who have had STN-DBS compared to medically treated patients [16]. Also several clinical studies have shown the clinical effectiveness of STN-DBS in improving Parkinsonian symptoms [22]. The benefit of STN-DBS on motor-function allowed a major improvement in all aspects of quality of life, especially the social functioning subscale, as already reported [23]. The present mathematical model confirms these results. According to this model the surgical action is cost effective confirming previously published clinical models and can be applied for patients with PD relatively early (e.g for moderate events) with economic benefits, quality of life and low risk.

## Conclusion and proposals

The clinical management requires the ability to predict the interplay between the natural history of diseases and the effects of intervening actions over time. Often, such predictions cannot be made with certainty and trade-offs have to be made between the expected benefits of current and future decisions. In our paper we presented how partially observable Markov decision processes can be used to formalize the management of a patient with Parkinson's disease, providing an explicit representation of the clinical states, the management strategy employed and the objectives of treatment. Therefore, the final aim of our research is to provide analyses and methods that can inform clinical studies. Our model crates a simulation of the actual disease monitoring processes. Consequently, our model should only be used complementary in the decision making process. As a fact, the implementation of our model in the real clinical practice, confirmed that it has the ability to sufficiently estimate the decisions taken by the clinicians. However, we don't suggest that our model should directly effect immediate changes in the treatment policy for patients with PD. Finally, we believe that our model provides an efficient and supplementary tool for determining a feasible set of treatment options to be examined in the clinical practice.

## Competing interests

The authors declare that they have no competing interests.

## Appendix 1

For the POMDP model we apply the following main performances index.

**Discounted-cost (DC)**:

(1)

**Average-cost (AC)**:

(2)

respectively, and in terms of the information vector *ρ*(t) by:

(3)

and

(4)

The equivalence, in the sense of equal optimal costs for each *π*(0) ∈ ∏, of the optimization problems defined using criteria (1) and (2), and similarly for problems specified using (3) and (4). Since the state of the patient is not known at time t, we will work with J_β_(*δ*, .) and J(*δ*, .).

Now we define:

(5)

Then, *V*_*β *_(*π*) is the total expected discounted cost accrued when an optimal policy is selected, given that the initial information vector is *ρ*, and future costs are discounted at rate *β*. It is well known that *V*_*β *_(*π*)is the unique solution of the functional equation:

(6)

When computing optimal policies in the infinite horizon case, we need only consider stationary policies [20]. A stationary policy is denoted by(*δ*)^∞ ^= (*δ*, *δ*, ...). Similarly, define:

*Optimal-average cost*: , *ρ *∈ Π. Then, g is the expected optimal average cost, and it satisfies the functional equation:

(7)

A strategy δ* (if it exists), is optimal if it is valid

(8)

For the average cost criterion, the limit of the expected average cost may not exist for some or all policies. Given the results above, naturally there has been considerable interest in finding conditions which guarantee the existence of a bounded solution (*g*, *h*) to the ACOE. It can be shown that a necessary condition for the existence of a bounded solution to the ACOE is that the following *uniform boundness *condition holds.

*(UB) There is a constant L *> 0 *such that*: [14]



*Theorem A*: *Suppose there is a bounded solution (g, h) to the ACOE. Then condition (UB) is satisfied with L = 2.span(h), where*



*Proof*. For the case when S, A are both finite, the result above can be inferred.

Various necessary conditions for the existence of a solution to the ACOE have been proposed in the literature.

Theorem B: If Assumption *(UB) *holds, then there exist a bounded, concave and continuous function *h*: Π → ℛ and an optimal cost *g *such that (*g*, *h*(.)) is a solution of the dynamic equation:



Proof. Ross [6]

Assumption (UB) can be checked to hold for the above problem, where we have a mixed observation possibility since both partial and complete observation can occur. Then there exist a bounded, concave and continuous function *h*: Π → ℛ and an optimal cost *g *such that (*g*, *h*(.)) is a solution of the dynamic equation:

, by theorem (B).

For the solution of the above equation we take the algorithm of Sondik [5] or the method of Goulionis et.al. [11].

## Authors' contributions

JG, carried out the model development and formulation, performed the statistical analysis and participated in the design and co-ordination of the study.

AV, conceived of the study, adjusted the protocols for clinical treatments decisions for Parkinson disease and participated in the model development and formulation.

All authors read and approved the final manuscript.
